# The use of an unsupervised learning approach for characterizing latent behaviors in accelerometer data

**DOI:** 10.1002/ece3.1914

**Published:** 2016-01-11

**Authors:** Marianna Chimienti, Thomas Cornulier, Ellie Owen, Mark Bolton, Ian M. Davies, Justin M.J. Travis, Beth E. Scott

**Affiliations:** ^1^ School of Biological SciencesUniversity of AberdeenTillydrone AvenueAberdeenAB24 2TZUK; ^2^ Marine Scotland ScienceScottish GovernmentMarine LaboratoryPO Box 101375 Victoria RoadAberdeenAB11 9DBUK; ^3^ RSPB Centre for Conservation ScienceNorth Scotland OfficeEtive House, Beechwood ParkInvernessIV2 6ALUK; ^4^ RSPB Centre for Conservation ScienceThe LodgeSandyBedfordshireSG19 2DLUK

**Keywords:** Accelerometer data, animal movements, behavioral classification, unsupervised learning

## Abstract

The recent increase in data accuracy from high resolution accelerometers offers substantial potential for improved understanding and prediction of animal movements. However, current approaches used for analysing these multivariable datasets typically require existing knowledge of the behaviors of the animals to inform the behavioral classification process. These methods are thus not well‐suited for the many cases where limited knowledge of the different behaviors performed exist. Here, we introduce the use of an unsupervised learning algorithm. To illustrate the method's capability we analyse data collected using a combination of GPS and Accelerometers on two seabird species: razorbills (*Alca torda*) and common guillemots (*Uria aalge*). We applied the unsupervised learning algorithm Expectation Maximization to characterize latent behavioral states both above and below water at both individual and group level. The application of this flexible approach yielded significant new insights into the foraging strategies of the two study species, both above and below the surface of the water. In addition to general behavioral modes such as flying, floating, as well as descending and ascending phases within the water column, this approach allowed an exploration of previously unstudied and important behaviors such as searching and prey chasing/capture events. We propose that this unsupervised learning approach provides an ideal tool for the systematic analysis of such complex multivariable movement data that are increasingly being obtained with accelerometer tags across species. In particular, we recommend its application in cases where we have limited current knowledge of the behaviors performed and existing supervised learning approaches may have limited utility.

## Introduction

The use of accelerometers has been recognized as a powerful method for studies of behavior and for accurate quantification of animal movements (Shepard et al. 2008; Wilson et al. 2008; Gómez Laich et al. 2009). A recent review emphasizes the already wide and rapidly accelerating use of accelerometers in studies of animal behaviors, in both aquatic and terrestrial habitats (Brown et al. 2013). Mammals represented 45.6% of over 120 species on which accelerometers have been deployed, followed by birds. However, most studies using accelerometer data to quantify animal behavior have required researchers to proceed with custom‐made analyses or involved manual identification of the different behaviors performed by the study species. The latest accelerometers are able to record at high rates, between 100 and 300 Hz (Bidder et al. 2014), producing a large amount of data and making the manual identification of behavioral patterns increasingly challenging (Resheff et al. 2014).

Recent approaches to accelerometer data analysis and latent behavioral class recognition have predominantly used supervised learning algorithms. Among supervised algorithms, the K‐Nearest Neighbor algorithm has been applied on species including Kangaroos, Camels, and Cormorants (Gómez Laich et al. 2009; Bidder et al. 2014). With this approach, previous knowledge about the different behaviors performed by the species is required. The researcher needs to manually label part of the behavioral database to create the training data necessary for the algorithm. Also, new methods and software based on supervised algorithms (Nathan et al. 2012; Resheff et al. 2014) have been developed where the identification of behaviors is customized depending on specific behavioral thresholds of the studied species (Gómez Laich et al. 2009). These approaches are not well‐suited to cases where a priori knowledge of the species' behavior is lacking as they may fail to identify important behavior types that are represented in the data but are neither detected nor expected by the researcher.

In addition, the small size of some species leads to difficulties in monitoring with other devices, such as cameras, and behaviors might be difficult to test and validate in controlled environments.

Among the few existing applications of unsupervised algorithms, Sakamoto et al. (2009) developed a tool able to analyse and classify accelerometer data automatically into several categories. Using the unsupervised algorithm k‐means, the software was able to identify general behaviors in cormorants and penguins (Sakamoto et al. 2009; Watanabe et al. 2012). However, while promising, this approach is limited in terms of the number and type of behavioral groups recognized and the amount of data that this particular clustering algorithm can handle (Sakamoto et al. 2009). In an increasing proportion of studies, we ideally require a method that can be effectively and efficiently applied to increasingly high volumes of data obtained and that can provide an accurate behavioral classification without an algorithm needing to being trained. In this study, we propose a new method for analysing accelerometer data and discerning between different behavioral modes which can handle large volumes of data and which does not require direct observations of the behavior of the animals.

To illustrate the capability of the method, we analyse data collected on two species of diving seabirds; common guillemot (*Uria aalge*) and razorbill (*Alca torda*). We focus on data collected during foraging trips, aiming to measure the different activities performed by these two species during their foraging activities. For this case study, we will demonstrate the potential of unsupervised learning algorithms to detect different behaviors in two diving species that use their wings for underwater propulsion and face evolutionary trade‐offs moving in both air and water (Kovacs and Meyers 2000).

Previous studies on the diet of both species have mainly be been based on observations of prey brought to the chick during the breeding season. Data collected in the North Sea concerning both self‐feeding and chick provisioning showed that both species take mainly sandeel, sprat, young Atlantic herring, whiting and cod (Rindorf et al. 2000; Mitchell et al. 2004). It is also necessary to consider that seasonal changes, environmental changes, and commercial fishing activities are likely to affect the proportion of fish species brought back for the chick or caught for self‐feeding (Anderson et al. 2014). Razorbills typically bring several fish back to the colony in their beak, while Common guillemots feeding chicks bring back a single fish (Thaxter et al. 2013). It is not known how many preys are captured during a single dive, and there is little knowledge of adult diet prior to laying. Information obtained from stomach flushing and fatty acid analysis indicated seasonal shifts in the diet and that prey diversity in common guillemot was higher than razorbills (Ouwehand et al. 2004; Owen et al. 2013).

It has been suggested that guillemots, having higher wing loading than razorbills (Pennycuick 1997; Hipfner & Chapdelaine, 2002; Thaxter et al. 2010), make greater use of the vertical dimension for foraging while razorbills make greater use of the horizontal dimension through flight (Thaxter et al. 2010). Guillemots perform longer and deeper dives than razorbills (Paredes et al. 2008; Thaxter et al. 2009) suggesting that the two species might use the water column differently and feed on prey distributed at different depths. During diving activity, both species alternate periods underwater with periods on the surface where they replenish oxygen in preparation for the next dive (Butler and Jones 1997). Dive shape, maximum depth, duration, and recovery periods on the surface can be different among species, meaning that each species can allocate its time in different ways depending on the foraging strategy performed (Elliott et al. 2008; Wilson et al. 2012). Despite information about the use of horizontal and vertical dimensions while foraging, to the best of our knowledge, no existing studies conducted on these species have looked at the use of both dimensions at the same time in order to gain a strong understanding of their behaviors and time/energy budgets in the water column and how they catch their prey. Thus, this study represents an ideal example where deploying accelerometers and then applying state‐of‐the‐art analytical tools can provide valuable information on foraging behaviors.

The primary aim of this study is to develop a generally applicable method for analysing accelerometer data that is able to independently (or automatically) identify common behavioral modes among individuals, as well as specific individual behaviors, in species moving in two or three dimensions (in our specific case, that move in three dimensions while foraging). Our secondary aim is to demonstrate its use on two species of diving seabird anticipated to have contrasting foraging behaviors aiming to clarify how these predators search for prey, as well as highlighting different movement patterns.

## Methods

We introduce the potential for Gaussian Mixture Models, which are often used to model the probability distribution of continuous measurements in data clustering approaches, data miming, pattern recognition, machine learning, and statistical analysis of high dimensional data (Biernacki et al. 2003). We first briefly describe the statistical approach (further details are available in the section below), before introducing the seabird data used within an example application.

### Maximum likelihood approach for fitting Gaussian Mixture Models

A powerful method for finding maximum likelihood solutions for mixture models with latent variables is called the *expectation‐maximization* algorithm, or *EM* algorithm (Dempster et al. 1977; McLachlan & Krishnan, 1997). The algorithm selects an initial setting for the parameters, denoted *θ*_*i*_. Then it alternates between two steps called *E step* and the *M step*.

Given a joint distribution *p*(*X, Z|θ*) over observed variables *X* and latent variables *Z*, governed by parameters *θ*, the algorithm maximizes the likelihood function *p*(*X|θ*) with respect to *θ*. Proceeding into the *Estep*, the values of the latent variables in Z are given by the posterior distribution *p*(*Z|X, θ*_*i*_). This approach considers the expected value of the log‐likelihood under the posterior distribution of the latent variable. In the *Mstep* the algorithm evaluates *θ*_new_, checking for convergence of either the log likelihood or the parameter values. If the convergence criterion is not satisfied, the algorithm returns to select the initial settings for the parameters recalculating *θ*_*i*_ (Bishop 2006).

In practice, the algorithm selects initial values for the means, covariance and mixing coefficients from the variables given (observed variables) and evaluates posterior probability distributions (latent variables, *Estep*). The probabilities are used in the *Mstep* to re‐estimate means, covariance and mixing coefficients weighted by the probabilities of each data point belonging to each cluster. Each update to the parameters resulting from an *Estep* followed by an *Mstep* increases the log likelihood function until convergence.

This method, being both unsupervised and able to deal with high dimensional data, represents an ideal solution for analysing the type of data collected with accelerometer tags.

### Data collection

**Figure 1:**
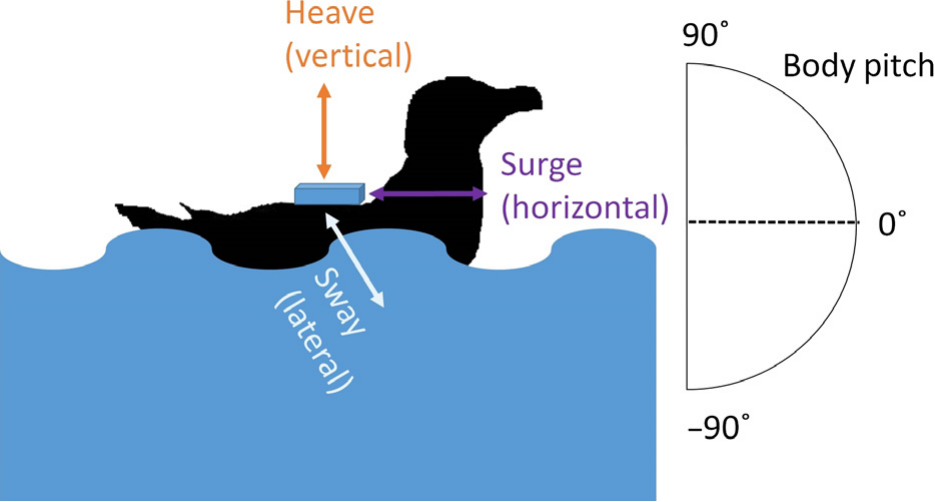
Example of the position of the accelerometer on a general seabird.

Data were collected in 2014 at two different locations in Scotland (UK), Colonsay (56°3′54″N, 6°24′21″W) and Fair Isle (59°22′55″N, 1°48′26″W). Three‐Axis Accelerometer tags (Axy‐Depth, TechnoSmArt, http://www.technosmart.eu/) were deployed in combination with GPS tags (Gt‐120, IgotU) and mounted using Tesa tape (Tesa, Extra Power) on the back of common guillemots (*Uria aalge*) and razorbills (*Alca torda*). The weight of the combination of the two devices plus the tape used was 25 g, <4 % of birds' body mass (Caccamise and Hedin 1985). The GPS tags were set to record each location every 100 sec, the accelerometer tags were set to record Pressure (millibar, accuracy of 0.5 millibar) and Temperature (˚C, accuracy of 0.1°C) at 1 Hz and the acceleration in the three dimensions (surge (horizontal) *A*_*h*_, sway (lateral) *A*_*l*_ and heave (vertical) *A*_*v*_) at 25 Hz (Fig. 1). Both devices were then retrieved after 2–4 days, when the animal was at the colony. Data from 2 common guillemots and 5 razorbills were collected respectively from Colonsay and Fair Isle (Scotland).

### Data preparation and variable selection

Pressure data were converted to depth (m), here on referred to as Depth, following the formula in UNESCO Technical Papers (Fofonoff and Millard 1983).

The formula followed accounts for compressibility (density). An ocean water column at 0°C (*t* = 0) and 35 PSU (*s* = 35) was assumed. The gravity variation with latitude and pressure is computed as:

(1) $$\begin{array}{cc} g\left(\frac{m}{{\text{sec}}^{2}}\right)=9.780318*[1.0+(5.2788*10^{-3}\; & \\ +2.36*10^{-5}*x)*x]+1.092*10^{-6}*p \end{array}$$

where *x *= [sin (latitude/57.29578)]^2^ and *p *= pressure (decibars). Latitude value was assumed to be the location of each seabird colony. *Depth* was then calculated from pressure:

(2) $$\begin{array}{cc} \text{Depth}\;(m)= & [(((-1.82*10^{-15}*p+2.279*10^{-10})\\ & *p-2.2512*10^{-5})*p+9.72659)*p]/g \end{array}$$

where *p *= pressure (decibars) g = gravity (m/sec^2^).

To calculate the orientation of the body angle, Pitch (*B*), and the Dynamic Acceleration in the three dimensions (surge *D*_*h*_ *,* sway *D*_*l*_ *,* heave *D*_*v*_), the signals were smoothed using a running mean of 1 sec for razorbills and 2 sec for guillemots to calculate first the Static Acceleration. The difference in the time window applied to the two species was due to the differences in the diving behavior between the two species (see Results sections). Static acceleration provided a measure of the body angle of the instrumented animals. Body Pitch and the Dynamic Acceleration were then calculated as follow:

(3) $$B={\text{tan}}^{-1}\left(\frac{S_{h}}{\sqrt{S_{l}^{2}+S_{v}^{2}}}\right)*\frac{180}{\pi }$$

(4) $$D_{h}=A_{h}-S_{h}$$

(5) $$D_{l}=A_{l}-S_{l}$$

(6) $$D_{v}=A_{v}-S_{v}$$

The measured values were corrected for imperfect device orientation by examining the Pitch value from each individual as it rested on the sea surface, assuming that this value was representing the true zero (Sato et al. 2003, Watanuki et al. 2003; Laich et al. 2008). All the signals were then standardized.

In addition to the variables commonly used in defining the different behavioral classes such as dynamic acceleration and body pitch (Shepard et al. 2008; Gómez Laich et al. 2009), a few more variables were derived from the data. Vertical speed (*V*_*s*_) was calculated as the change in depth per second. The amplitude of the signal of the heave *Amp*_*v*_ and the variance of the difference *B*_var_ of the pitch, were calculated as the standard deviation over a running mean of 5 sec for the guillemots and 10 sec for the razorbills (Table 1). It was assumed that different behaviors might be detectable in different time windows and by combining variables. Finding the appropriate variables and parameters was an iterative process which depends on the data and tasks at hand. This involved testing the EM with different combinations of variables and parameters. For example the variables *Amp*_*v*_ and *B*_var_ were tested across window sizes of 3, 5, and 10 sec each.

**Table 1:** List of the variables obtained and calculated from the accelerometer data

Parameter	Label	Definition
Acceleration recorded from the accelerometer	*A*_*h*_, *A*_*l*_, *A*_*v*_	Surge (horizontal), Sway (lateral), and Heave (vertical)
Depth	*Depth*	
Vertical speed	*V*_*s*_	Change in depth every second
Static acceleration	*S*_*x*_ *, S*_*y*_ *, S*_*z*_	Surge, Sway and Heave
Dynamic acceleration	*D*_*x*_ *, D*_*y*_ *, D*_*z*_	Surge, Sway and Heave
Pitch	*B*	Vertical orientation of the body angle
Amplitude	*Amp*_*v*_	Standard deviation over a running mean of 5 sec for the guillemots and 10 sec for the razorbills
Variance of the pitch	*B*_var_	

### Dive analysis

Depth data were analysed using MTDive (MultiTrace Jensen Software). A dive was deemed to have occurred when the maximum depth was ≥1 m. Bottom time was calculated checking for points of inflection in the depth profile.

### Modeling approach: behavioral characterization

The proposed approach followed an iterative process consisting of considering the general knowledge of the environments where the data were collected, the general behaviors known about the study species (fly, float and dive) and the statistical properties of the variables calculated. For example, the study species perform constant flapping while flying, producing high value for acceleration in the vertical dimension. The calculation of the amplitude *Amp*_*v*_ highlighted the consistency of such behavior over a time window of 5 sec for guillemots and 10 sec for razorbills. The effect of every new variable on the partition performed by the *EM* algorithm was checked every time that a new variable was calculated and added to the list of variables used in the model.

To simplify the analytical procedure, since our primary aim was to clarify behavioral states associated with foraging when no additional information is available, we used the depth data from the accelerometers to divide the data for each animal into time spent above and below water.

For the underwater data, the EM was run for different numbers of latent behavioral classes. The selection of the best model was made by observing the type of partition that the algorithm produced and the number of clusters that could be ecologically explained. The variables selected for these runs were *Amp*_*v,*_ *B*_var_ and the standardized channels of *V*_*s*_, *D*_*h*_ *, D*_*l*_ *, D*_*v*_ and *B* for both species.

For the subset of the data containing the activities above water, it was not our aim to observe all potential behaviors that these species are able to perform above water. The observation of the two variables *Amp*_*v,*_ and *D*_*v*_ highlighted differences between the animal being in motion or stationary, so it was a‐priori decided to focus on the main activities that could be performed such as flying, floating and sitting on land. The EM was configured to recognize three main latent behavioral classes, corresponding to three general activities: high activity while flying and flapping, medium activity while floating or walking at the colony, and null activity, corresponding to the animal sitting at the colony or floating on a calm sea surface. The variables used for this run were *Amp*_*v*_ and the standardized channels of *D*_*h*_ *, D*_*l*_ *, D*_*v*_ *,* and *B*.

Accelerometer data were matched with the GPS positions and distances from the colony were then calculated to observe how the classification above water was distributed on a spatial scale and whether activities occurred at the colony or at sea. The GPS position of the colony was represented by the first GPS position in the data, the full R code for calculating the distances is included in Data S3. Results are presented for two of the activities performed above water: medium and low activity.

To observe individual variability and general species behaviors, the algorithm was run at both individual and species level. Where individuals were combined by species, due to the differences in the total time of deployment among both individuals and species, the datasets of the two common guillemots were sampled so to obtain 15 h of deployment from each animal. The datasets of the five razorbills were sampled to obtain 22 h of data from each individual. The two common guillemots were labeled as COGU_1 and COGU_2 and the five razorbills RAZO_1, RAZO_2, RAZO_3, RAZO_4, and RAZO_5. Species were labeled as COGU and RAZO.

To observe the structure and the order of the behavioral changes classified by the *EM* algorithm, we calculated transition probability matrices (Bishop 2006). For simplicity, a behavior was deemed to occur if consistent for a minimum of 1 sec, so the partition performed by the *EM* was smoothed using a running mean of 1 sec. Given a behavioral state *Z* at time *t* (*Z*_*t*_), we looked at the behavioral state at the previous time step (*Z*_*t‐1*_) and calculated the probability of staying within the same state or switching between different states. The transition probability matrices were calculated for each species, pooling together the results obtained from the runs on all individuals.

Data preparation and analysis were performed in R version 3.0.2 (R Core Team 2013). The EM analysis was performed using the R package *RMixmod* (Biernacki et al. 2006). For brevity, results are shown only for two of the combination of variables used in the analysis, *D*_*v*_ and *B* on both common guillemots and for one razorbill as examples (RAZO_3). The partition performed also on other variables such *V*_*s*_, *D*_*h*_ *, D*_*l*_ *,* for both common guillemots and for one razorbill as examples (RAZO_3) are shown in the Data S1 and S2. The R code used for the calculation of the variables and the analyses is also shown in the Data S3.

Groups of behavioral states were classified as UW when an individual was underwater, and AW when it was above water. Both groupings are individual and species specific and each behavioral state is denoted with a number (i.e. UW1, UW2…). The colors in the plots and further explanation in the results section will highlight common behavioral states for comparison across individuals and species.

## Results

### Dive analysis

**Figure 2:**
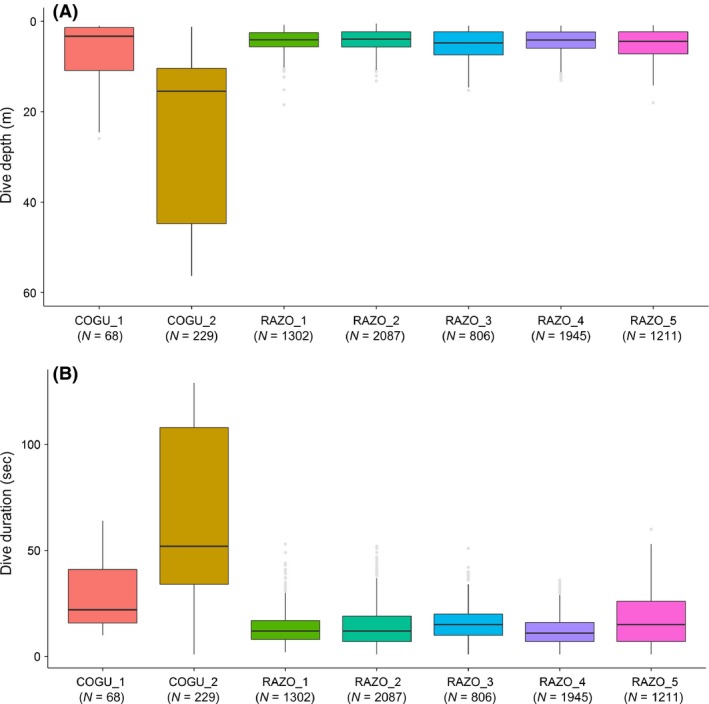
Dive depth (A) and duration (B) performed by two common guillemots and five razorbills equipped with accelerometers. *N* = number of dives.

The two common guillemots (COGU) performed deeper and longer dives than the five razorbills (RAZO), (common guillemot, depth (m) mean = 43.56, SD = 18.52, duration (sec) mean = 57.35, SD = 37.56; razorbill, depth (m) mean = 4.49 SD = 2.48, duration (sec) mean = 14.22, SD = 9.02, Fig. 2A,B). The frequency of dives was lower in common guillemots compared to razorbills (4 dives/h and 17 dives/h respectively).

### Classification of groups of animals

**Figure 3:**
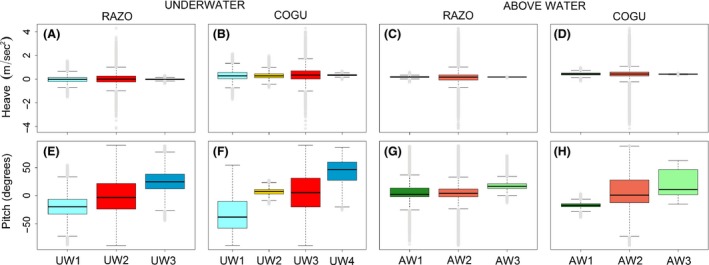
Behavioral partition performed by the unsupervised algorithm Expectation Maximization on razorbills (RAZO) and common guillemots (COGU), underwater (UW) and above water (AW). The dynamic acceleration performed in the vertical axis (Heave) and the vertical orientation (Pitch) are shown. Colors represent the different behavioral states recognized by the EM algorithm, the same colors correspond to the same behaviors. RAZO (A, E, C and G): UW1 = Descending phase, UW2 = Searching/Catching phase, UW3 = Ascending phase. COGU (B,F, D and H): UW1 = Descending phase, UW2 = Deep searching phase, UW3 = Catching phase, UW4 = Ascending phase. AW1, AW2 and AW3 = medium, high and low activity above water corresponding to floating on the sea surface, flying and standing at the colony.

**Figure 4:**
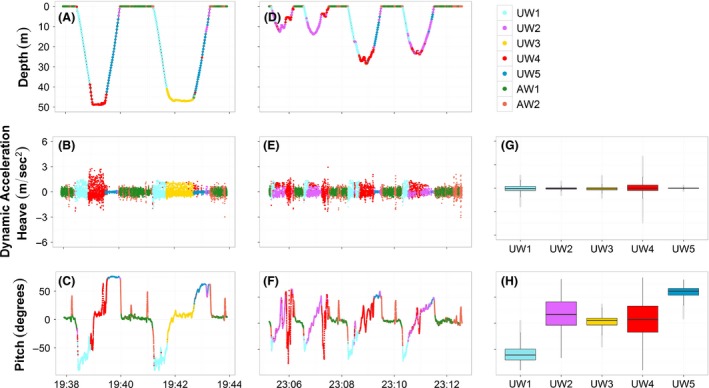
Example of the latent behavioral classes' recognition performed by COGU_2 in both deep (A, B, C) and shallow (D, E, F) dives. A and D represent the diving depth (m), B, E and G the dynamic acceleration performed in the vertical axis (Heave), C, F and H the vertical orientation (Pitch). Colors represent the different behavioral states recognized by the EM algorithm, the same colors correspond to the same behaviors. Underwater, UW1 = Descending phase, UW2 = Shallow searching phase, UW3 = Deep searching phase, UW4 = Catching phase, UW5 = Ascending phase. Outside water, AW1 = floating, AW2 = flying/flapping.

Based on the partition performed by the *EM* algorithm it was possible to recognize different behaviors among the two species both underwater and above water (Fig. 3). The classification performed on the combination of the two common guillemots divided the underwater data into four main behavioral classes: descending phase, deep searching phase, chasing/catching events, and ascending phase (Fig. 3B,F). The descending phase (mean ± SD, Pitch (degrees) −36.30 ± 27.52, Heave (m/s^2^) −0.0084 ± 0.43, Fig. 3B,F, UW1) was characterized by negative pitch angles accompanied by moderate values in the heave acceleration reflecting the stroking movements downwards. During the deep searching phase (Pitch (degrees) 4.11 ± 5.74, Heave (m/s^2^) −0.0042 ± 0.37, Fig. 3B,F, UW2) the animal was mainly in the deep part of the water column moving horizontally (Example on COGU_2, Fig. 4A‐C). This state was present mainly in deep dives. During chasing/catching events (Pitch (degrees) 0.43 ± 36.43, Heave (m/s^2^) ‐ 0.03 ± 0.63, Fig. 3B and F, UW3) the guillemots performed fast and sharp changes of their orientation in the water column, this state showed the highest variance in all variables among the states classified underwater (Data S1). While ascending in the water column (Pitch (degrees) 42.78 ± 20.42, Heave (m/s^2^) −0.015 ± 0.083, Fig. 3B,F, UW4) the animal's acceleration measured in the vertical dimension was very small compared with the other states, indicating the animal being passively pushed up by the pressure of the water column.

**Figure 5:**
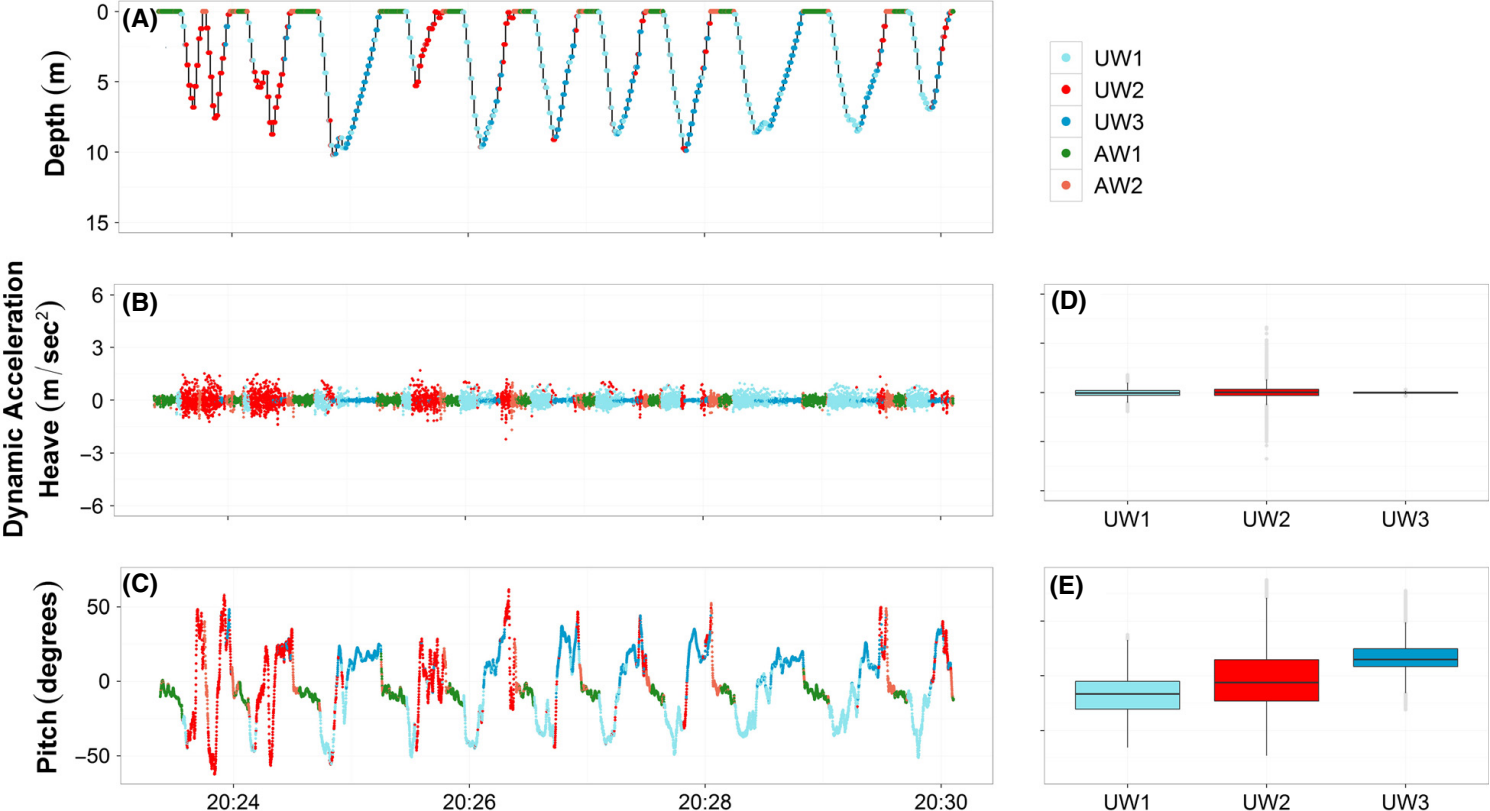
Example of the latent behavioral classes' recognition performed by RAZO_3. A represents the diving depth (m), B and D the dynamic acceleration performed in the vertical axis (Heave), C and E the vertical orientation (Pitch). Colors represent the different behavioral states recognized by the EM algorithm, the same colors correspond to the same behaviors. Underwater, UW1 = Descending phase, UW2 = Searching/Catching phase, UW3 = Ascending phase. Outside water, AW1 = floating, AW2 = flying/flapping.

The run performed on the combination of the five razorbills clearly divided the underwater data in three main behavioral classes: descending phase, chasing/catching events and ascending phase (Fig. 3A,E). As for the common guillemot, during the descending phase the animal was mainly facing downwards while descending in the water column (Fig. 3A,E, UW1, Pitch (degrees) −19.75 ± 17.65) with an acceleration in the vertical dimension indicating the effort of the movement in this phase (Heave (m/s^2^) −0.016 ± 0.30). During chasing/catching events (Fig. 3A,E, UW2), the animals made fast and sharp changes in their orientation in the water column (Example on RAZO_3, Fig. 5C‐E) producing high variability in both the Pitch and Heave (respectively −0.42 ± 31.97, 0.02 ± 0.47) channels. While ascending in the water column e (Fig. 3A,E, 3), the animal was mainly facing upwards (Pitch (degrees) 26.32 ± 19.45). The acceleration in the vertical dimension (Heave (m/s^2^) −0.012 ± 0.067) was very small compared with the others in the other states, indicating the animal being pushed up by the pressure of the water column.

**Figure 6:**
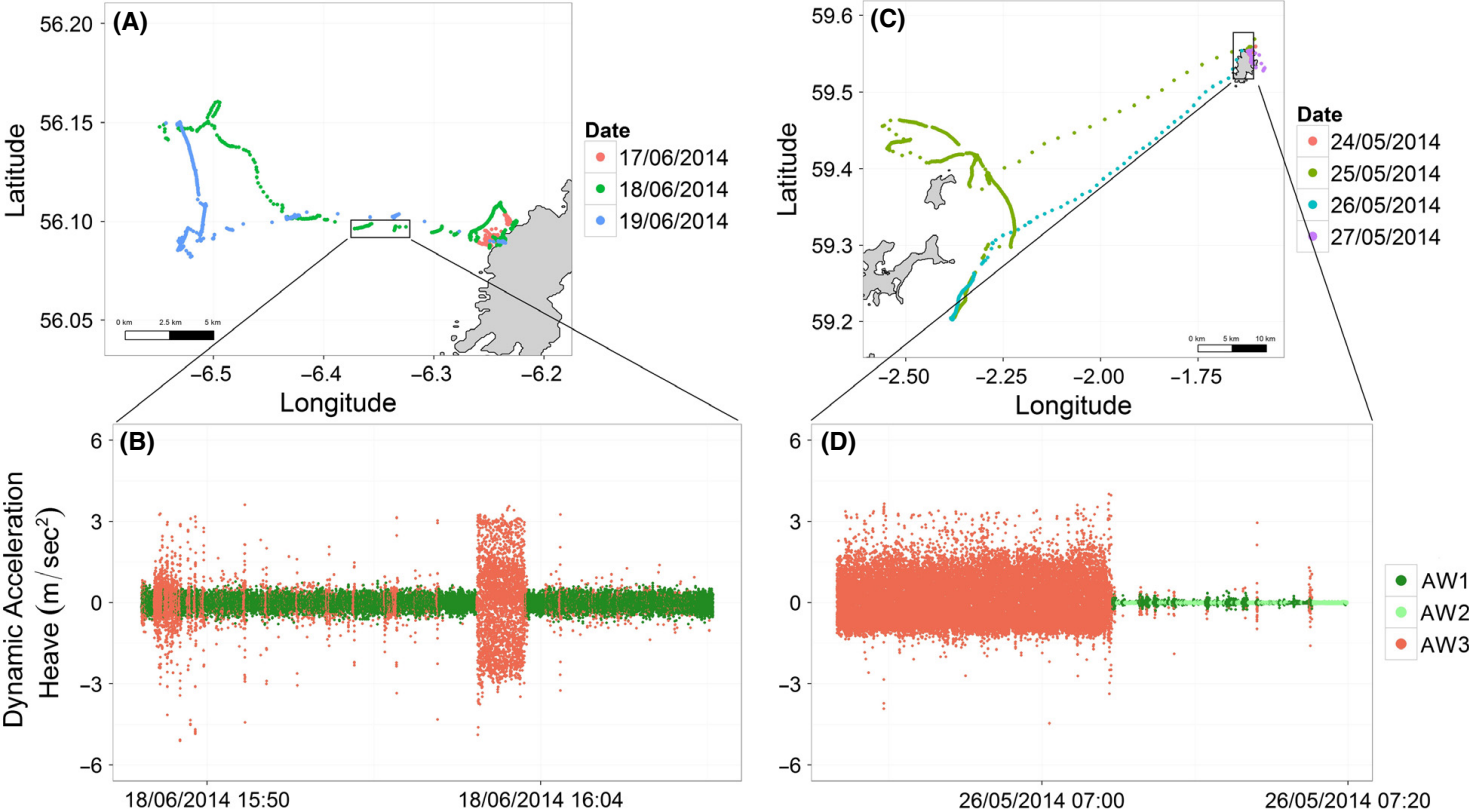
Example of above water activity in both common guillemot (A and B) and razorbill (C and D). A and C represent GPS tracks and B and D sections of the dynamic acceleration recorded in the vertical dimension (heave) corresponding to the GPS positions highlighted in the boxes. OW1 = floating on the sea surface/medium activity at the colony, AW2 = flying/flapping, AW3 = standing sitting, low activity.

**Figure 7:**
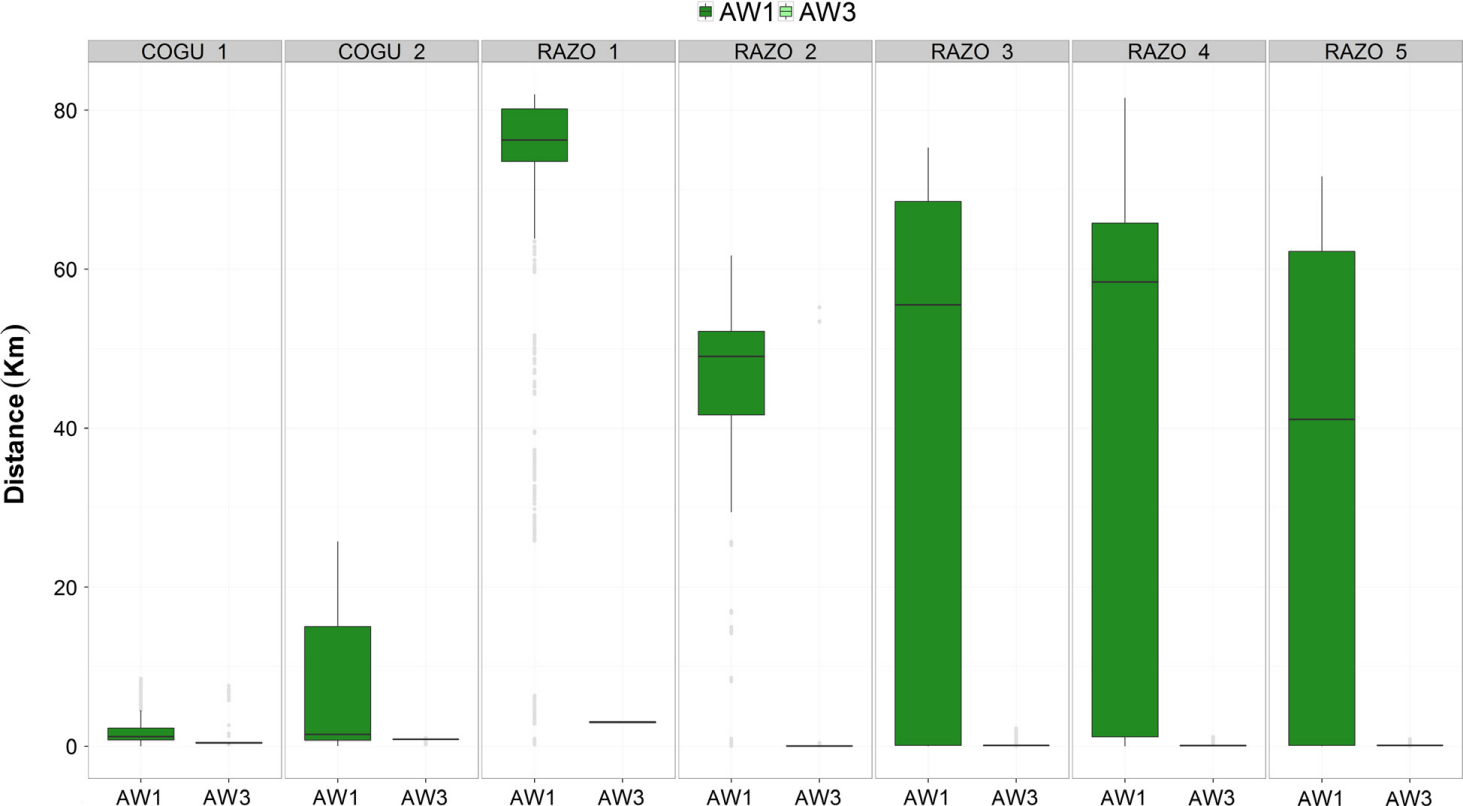
Distribution of behavior AW1 (medium activity, i.e. floating, walking at the colony) and AW3 (low activity, i.e. still at the colony) in relation to the breeding colony.

The three behavioral states labeled above the water surface represented three general activities that both species can perform during a foraging trip (Fig. 6). These states were called medium, high, and low activity (AW1, AW2, and AW3) as they were constant among all animals, both individually and as species group (see individual results below). These three types of behaviors were identified similarly in both species. State AW1 represented mainly the animal floating on the water surface but also when walking at the colony, as suggested from the analysis of the distances to the colonies (Fig. 7). Depending on the ocean condition, this state could have a larger variance in the Pitch channel, as shown for the razorbills (mean Pitch (degrees) 9.7 ± 19.86, Fig. 3G, AW1). However, the signal recorded in the Heave channel clearly showed a low level of movements (Heave, mean ± SD, 0.001 ± 0.10, Fig. 3). State AW2 represented mainly the flying and flapping activity performed while the animal was travelling to and back from the foraging area, or during short high activity phases such as flapping on the water column or at the colony. This state resulted in the highest variance in both Pitch and Heave among the states classified outside water (Fig. 6C,D, AW2). State AW3 represented mainly the animal not performing any type of movement, while floating on calm water surface or standing/sitting at the colony (Fig. 7).

### Classification of individual animals

**Figure 8:**
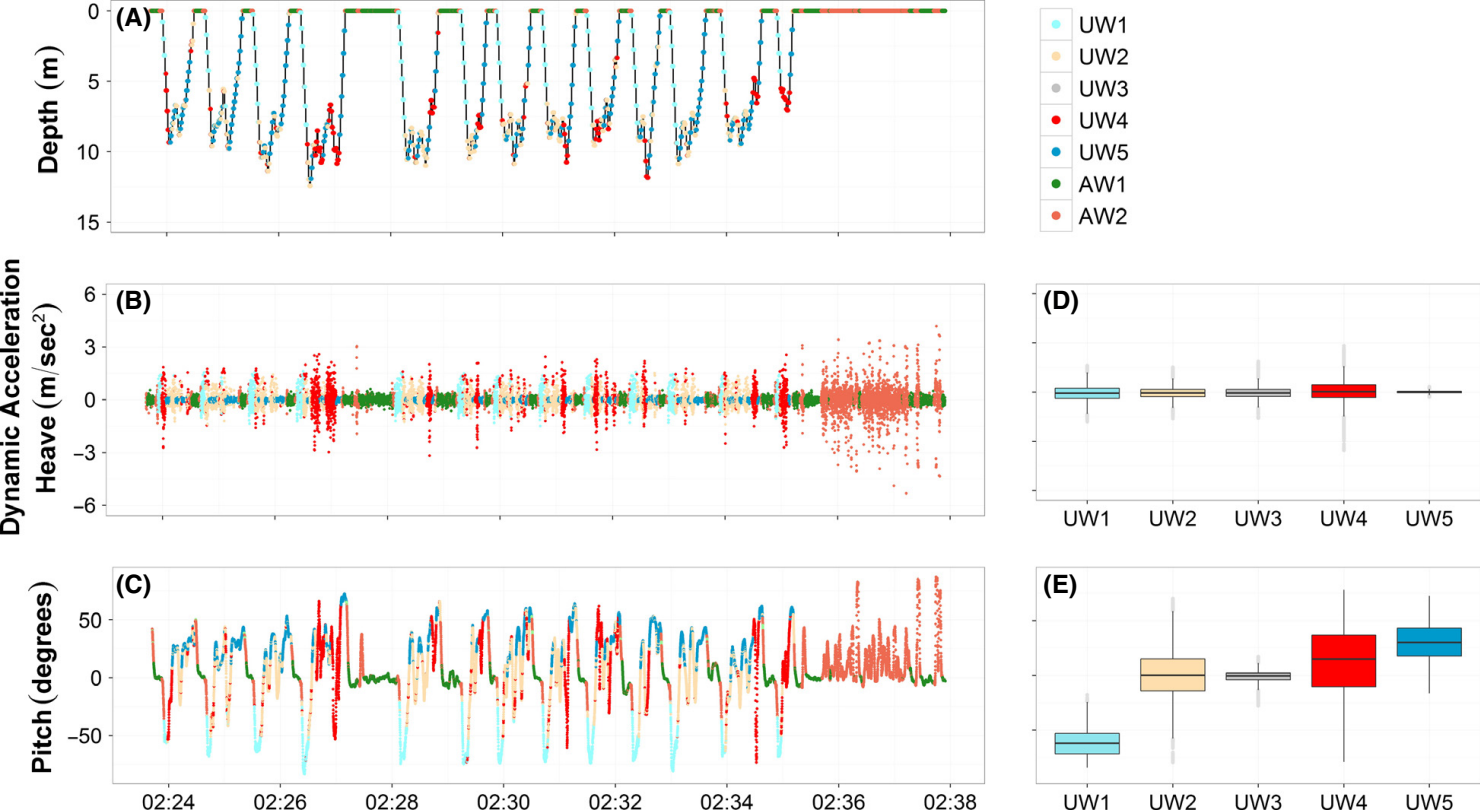
Example of the latent behavioral classes' recognition performed by COGU_1. A represents the diving depth (m), B and D the dynamic acceleration performed in the vertical axis (Heave), C and E the vertical orientation (Pitch). Colors represent the different behavioral states recognized by the EM algorithm, the same colors correspond to the same behaviors. Underwater, UW1 = Descending phase, UW2 = Shallow searching phase, UW3 = Shallow flapping, UW4 = Catching phase, UW5 = Ascending phase. Outside water, AW1 = floating, AW2 = flying/flapping.

Based on the partition returned by the *EM* algorithm performed on the underwater dataset, it was possible to recognize up to five behaviors in individual common guillemots and three behaviors in individual razorbills, highlighting differences among species. The analysis on individual common guillemots highlighted extra behaviors such as shallow searching and shallow activities. The analysis of COGU_1 classified five different behaviors, where three (descending, chasing/catching and ascending) were consistent with those classified in the group analysis (Fig. 8). The five behaviors were classified as descending, shallow searching, shallow activity, chasing/catching, ascending. The analysis of the COGU_2 also classified five different behaviors: descending, shallow searching, deep searching, chasing/catching, ascending, where four (descending, deep searching, chasing/catching, ascending) were consistent with the four classified in the group analysis (Fig. 4). The three behaviors classified in each razorbill were consistent with those resulting from the analysis on the entire group. The three behaviors were classified as previously, Descending phase, Searching/Catching phase, and Ascending phase (Fig. 5). The transition probability matrices clearly showed the structure of the changes between the behavioral changes in both species (Tables S1 and S2). For the underwater movements in particular, common guillemots showed a general sequence of behaviors made of: Descending (UW1), Searching (UW2 or UW3), Chasing/Catching (UW4) and Ascending (UW5). The probability of switching between Descending (UW1) and Ascending (UW5) was negligible, as was the probability of switching between the two searching phases UW2 and UW3 in COGU_2. By contrast, razorbills showed high probabilities of switching between the three states detected underwater: Descending (UW1), Chasing/Catching (UW2) and Ascending (UW3). The probability of switching from Descending (UW1) to Chasing/Catching (UW2) was lower than the probability of switching from Ascending (UW3) to Chasing/Catching (UW2).

## Discussion

Accelerometers have the potential to provide a wide range of detailed information on animal behavior and physiology. However, inferring behavioral models from the complex multidimensional data that accelerometers yield is crucial for realizing the potential and methods capable of informing this inference have just begun their development (Jonsen et al. 2013). A suite of statistical tools, including mechanistic multistate movement models (Morales et al. 2004), Hidden Markov Models (Langrock et al. 2012), Markov switching autoregressive models (Pinto and Spezia 2015) and State‐Space Models (Jonsen et al. 2005; Bestley et al. 2013; Bailey et al. 2014) have already begun to be applied to the analysis of time‐series movement data resulting from GPS, data storage tags and telemetry data. Mobile marine predators forage in a three dimensional environment (Shiomi et al. 2010) and more complex state‐space models have started to integrate one‐dimensional diving traces with two‐dimensional horizontal movement tracks (Bestley et al. 2015), highlighting the importance and the difficulty of combining multiple dimensions and variables when animals move and forage in more than two dimensions. Here, we have demonstrated that unsupervised learning algorithms can provide an important additional tool for analysing accelerometer data. We suggest that this new approach will be of particular utility for the many cases when it is not possible to use or collect any additional measurement or data as proxies for the classification of the animal's behavioral modes.

Using two species of seabird as a case study, we have demonstrated that the expectation maximization (*EM*) algorithm, effectively and efficiently classifies different behaviors in both species at both group and individual levels. In addition, the flexibility of this approach highlighted differences, similarities and new insights in the underwater foraging strategies of the two study species. We will first discuss the specific results of our case study before highlighting opportunities and challenges associated with using this unsupervised learning approach in comparison with the currently more frequently used supervised learning algorithms.

### The behavior of razorbills and guillemots foraging underwater as revealed by the unsupervised learning algorithm

The approach was tested on two different species known to feed on similar prey and for behaving differently underwater, but knowledge was lacking on exactly how the behaviors differed. Number of dives performed, time spent underwater, depth reached (Fig. 2) and the partition provided by the *EM* algorithm (Figs 4, 5 and 8) clearly distinguished where and how the individuals behave differently, highlighting differences in movements, foraging strategies and suggesting that the two species might use the vertical dimension differently (Thaxter et al. 2010).

Razorbills mainly fed in shallow water, probably feeding on shallow fish aggregations and performing three different activities: descending, chasing/catching, and ascending (Fig. 5 and Data S2). Common guillemots performed both shallow and deep dives, showing more flexible underwater movements and performing distinctive searching activities, not detected in razorbills. Differences in movement patterns performed by the two common guillemots (Figs 4 and 8 and Data S1) can be attributed to the fact that the two individuals foraged in two different part of the water column (COGU_1 mean depth 6.9 ± 6.658 m, COGU_2 mean depth 26.03 ± 18.664 m). In guillemots buoyancy decreases with increasing depth changing from positive to negative at about 60–70 m (Lovvorn et al. 2004). Foraging animals aim to maximize their foraging efficiency (Pyke et al. 1977; Halsey and Butler 2006) and the combination of their physiology and morphology (Butler and Jones 1997) and the distribution and abundance of resources, determine different types of movement (Giuggioli and Bartumeus 2010; Bartoń and Hovestadt 2012). The effect of the pressure due to the depth, sea bed, prey distribution and type of prey caught are suspected to be the main contributors to different type of movements and dive profiles (Elliott et al. 2008; Cook et al. 2010), making individuals perform different orientations and types of movements. The deep searching behavior (Fig. 4, UW3), for example, resulted in a lower variance in Pitch and Heave distributions compared with the shallow searching (Fig. 4 UW2, Fig. 8 UW2).

In both species the chasing/catching phase was characterized by a high variance in pitch angles and high peaks in the heave, surge and sway acceleration (Data S1 and S2) reflecting the mechanical effort of the animal when driving at prey both in the middle of the water column and near the sea bed. Prey such as squid and fish schools can be taken by birds from below, diving underneath and rapidly swimming up (Wilson and Duffy 1986; Crook and Davoren 2014) explaining the fast and sharp changes of orientation in the water column and the high peaks in the three dimensions (Zimmer et al. 2011).

### Gaining insights using a transition probability matrix approach

Comparing and contrasting the foraging behaviors of different species (or different populations within a species) will be a major area of research interest in the coming decade, as costs of tracking technologies reduce and more individuals can be sampled. Developing approaches that facilitate this comparison will be important and here we have used a transition probability matrix approach. The sequence of behaviors illustrated in the transition probability matrices provides a very clear means of identifying and quantifying the different behavioral strategies undertaken by the two species underwater. At least within our small sample of individuals, this approach reveals that our common guillemots always switched from descending to searching before ascending. In contrast, all of the razorbills in this study showed high probabilities of switching between underwater states, in particular between chasing/catching events and ascending phases. The behavioral switching that we observe in the razorbills suggested similarity with the “rush and grab” behaviors shown in other seabirds species (Wilson and Duffy 1986; Wilson et al. 2002). We suggest that the development and consistent application of approaches such as the transition matrix used here will play a vital role in determining key similarities and differences between species, or between populations.

### Multiscale foraging behavior can be revealed by joint deployment of GPS and accelerometers

While foraging, marine predators display movement patterns at multiple spatial and temporal scales and they are assumed to match the spatial structure of prey aggregations (Fauchald et al. 2000; Regular et al. 2013). The combination of high frequency GPS and Time Depth Recorders (TDRs) allows the study of both vertical and horizontal fine‐scale foraging behavior (Dragon et al. 2012; Evans et al. 2013) permitting a better understanding of behavioral responses to the variability in prey distribution. We fitted our birds with a combination of GPS and accelerometers and the data this provides (see Fig. 6) has the potential to reveal how very fine scale behaviors (such as those analysed in this study) related to larger spatial scales of behavior. Notably, the combined use of instruments adds new knowledge about the behavioral modes performed above water, allowing a reliable quantification of time spent flying, floating and at the colony. The increase in precision of use of time and space begins to allow a much greater understanding of exactly where and how marine predators start to search for prey and how the foraging behavior may vary both spatially and temporally. Furthermore, increased understanding of the multiscale foraging behavior can yield important information on how the quality of habitat patches and resources vary in time, and can enable insights into the potential effects of habitat changes (Hussey et al. 2015). As we gain data from an increased sample of individual razorbills and guillemots we will prioritize the joint analysis of GPS and accelerometer data.

### Energetics

Accelerometers have allowed the investigation of the biomechanics of diving birds in great detail (Yoda et al. 2001; Sato et al. 2002; Watanuki et al. 2003; Elliott et al. 2012) showing high correlation between overall dynamic body acceleration (ODBA) and the rate of oxygen consumption (VO2) in both great cormorants *Phalacrocorax carbo* (Wilson et al. 2006) and humans (Halsey et al. 2008). The dynamic component of body acceleration has been used as an index of mechanical power to quantify energy expenditure of different behavioral activities (Wilson et al. 2006; Elliott et al. 2012). Relating the OBDA expressed in each behavior/activity highlighted here, with the type of movement and type of dive, will have the potential to generally inform the variation in energetic costs of different dives and foraging trips. This new information can be used in future movement models by matching the different movement modes and movement variables such as speed, orientation and energy costs. Therefore, with the new approach presented here, it will be possible to acquire a more complete picture of the mechanisms underlying movement patterns as well as likely responses to spatial heterogeneity and habitat modification.

### Variable selection

The variables and parameters used in this work were calculated from the basic recordings of accelerometer tags delineating an approach transferable to other species and study systems. Using the methods presented here will make the future analyses of this type of data easier and more flexible. Importantly, the selection of the different variables to be included in the EM algorithm played a critical role in the analysis. The calculation of integrated variables such as vertical speed, the amplitude, and variance of the different signals, helped the discrimination of distinctive behavioral phases. For example, flight/flapping and chasing/catching prey underwater were behavioral modes both characterized by high peaks in the amplitude of the Heave channel and the calculation of these integrated variables helped highlighting such behaviors. We expect the use of these integrated variables to have the potential to also improve the performance of conventional supervised analyses. Depending on the species and the systems considered these types of integrated variables might be more useful than just using the raw output values of the accelerometer tags (Wang et al. 2015).

### Relative merits of the unsupervised approach

Both supervised and unsupervised approaches have advantages and disadvantages. In supervised learning, the model defines the partition of the observations depending on the input set called “labels”, where each cluster has been defined. In unsupervised learning, instead, it is assumed that the observations are governed by latent variables, so no input set exists and the model aims to find the hidden structure in the observations (Bishop 2006). From an ecological point of view, supervised algorithms allow the identification of known behaviors for which there is a training data set. However, this approach does not readily allow the identification of new and unknown behaviors. Unsupervised algorithms, instead, might be better suited for the type of data where behaviors cannot be observed and will allow the classification of unknown behaviors into different categories.

Estimating the frequency of events being mislabeled, and the accuracy of unsupervised approaches, is quite difficult when a validation dataset of “correct labels” is not available. It is possible to obtain an indication of mislabeling events by comparing the labels with a “more correct” set of labels obtained by smoothing the classification. Flexible smoothing functions that consider the type of behavior performed and the likelihood of having such behavior for a sufficient length of time can approximate a validation dataset. The comparison between original and smoothed labels will make it possible to indicate the accuracy of the unsupervised learning methods. In the current work, the transition probability matrix was calculated from a smoothed classification. Here, we arbitrarily chose to smooth over any behavior that was not consistent for more than 1 sec in order to observe sequences of behaviors. As already stated, further work in this direction is needed in order to better quantify mislabeling and uncertainty.

## Conclusions

The general challenge of identifying the mechanisms underlying ecological patterns is particularly relevant for movement research (Holyoak et al. 2008). When deployed with other sensors, accelerometers can provide a wide range of detailed information on the surrounding environment, physiology, and animal behavior (Johnson and Tyack 2003; Wilson et al. 2008). However, it might be challenging to record such information when monitoring longer periods, migrations, and winter habitats in both marine and terrestrial environments. The size of the study species might not always allow deployment of multiple data storage tags and it is not always possible to have direct observations of the behavior of animals in controlled environments for validation purposes. Such cases, as the one proposed here, emphasize the need for approaches able to automatically recognize the different behavioral pattern. The novel approach presented in this study is based on the combined use of the unsupervised learning algorithm *Expectation Maximization* and the calculation of new integrated variables. This approach can detect latent behavioral states at both group and individual level, highlighting key behavioral modes of two different species. Depending on the environment and species considered, integrated variables can highlight different types of behaviors allowing the user to avoid setting species specific thresholds and to generalize the method and not bias model outputs. Integrated variables are a fundamental aspect when developing new methods able to analyse such complex movement data collected both in the terrestrial and marine environment.

## Conflict of Interest

None declared.

## Supporting information


**Data S1.** Example of the latent behavioural classes' recognition performed in COGU_1 and COGU_2 underwater.

**Data S2.** Example of the latent behavioural classes' recognition performed in RAZO_3.

**Data S3.** R code used for the analysis of the accelerometer data.

**Table S1.** Transition probability matrix of the behavioural states classified in the 5 razorbills.
**Table S2.** Transition probability matrix of the behavioural states classified in the 2 guillemots.
