# Supplementary material for: The use of an unsupervised learning approach for characterizing latent behaviors in accelerometer data
Source: Ecol Evol. 2016 Jan 11;6(3):727–41. doi: 10.1002/ece3.1914 (PMC4739568; doi:10.1002/ece3.1914)
Supplement: Supplementary file 1 — Data S1. Example of the latent behavioural classes' recognition performed in COGU_1 and COGU_2 underwater. [file ECE3-6-727-s001.docx]

Supplementary Material 1


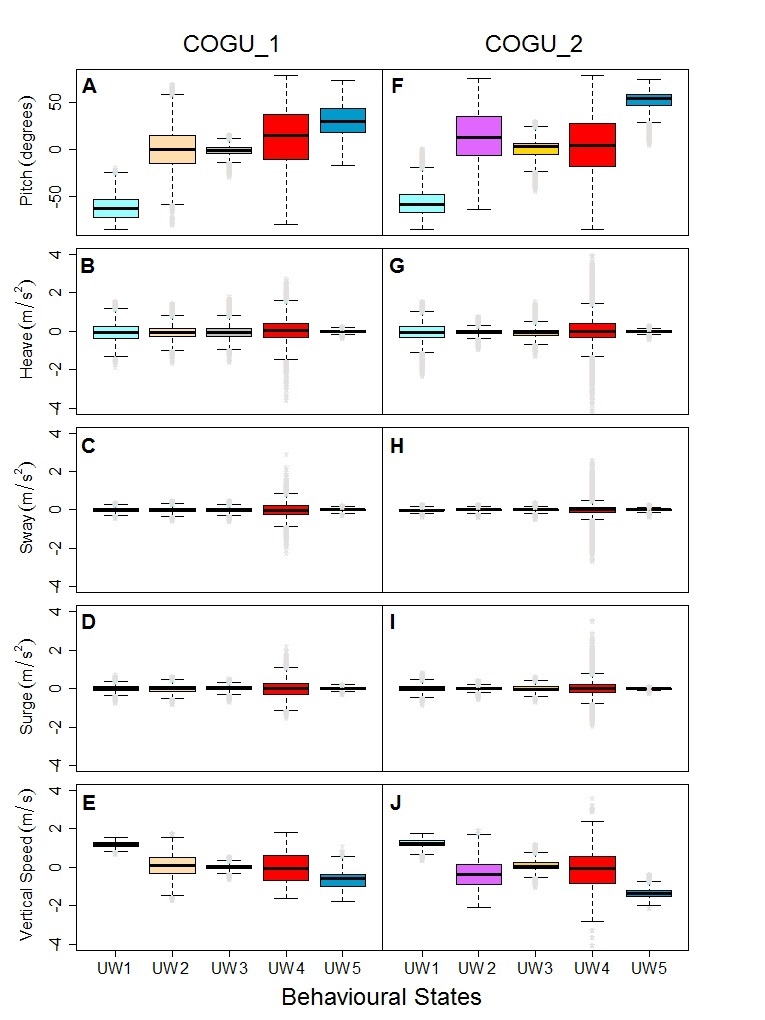


Example of the latent behavioural classes’ recognition performed in COGU_1 and COGU_2 underwater. **COGU_1:** UW1 = Descending phase, UW2 = Shallow searching phase, UW3 = Shallow flapping, UW4 = Catching phase, UW5 = Ascending phase. **COGU_2:** UW1= Descending phase, UW2 = Shallow searching phase, UW3 = Deep searching phase, UW4 = Catching phase, UW5 = Ascending phase
