# Supplementary material for: The use of an unsupervised learning approach for characterizing latent behaviors in accelerometer data
Source: Ecol Evol. 2016 Jan 11;6(3):727–41. doi: 10.1002/ece3.1914 (PMC4739568; doi:10.1002/ece3.1914)
Supplement: Supplementary file 2 — Data S2. Example of the latent behavioural classes' recognition performed in RAZO_3. [file ECE3-6-727-s002.docx]

Supplementary Material 2


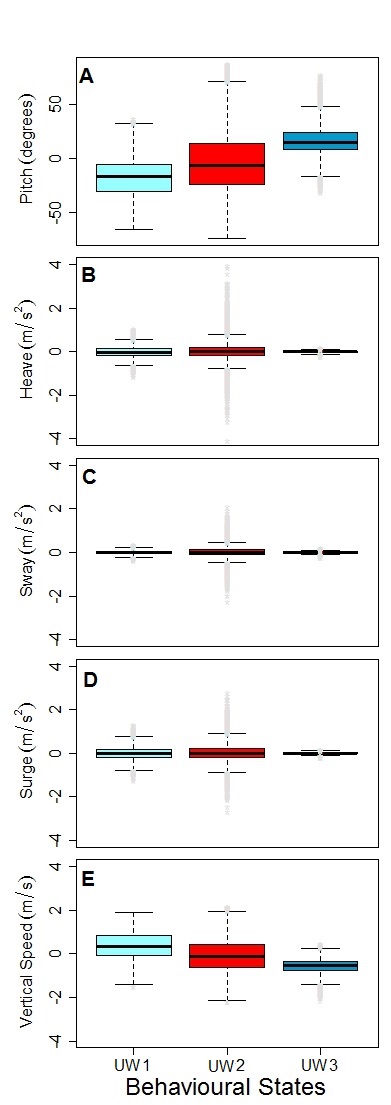


Example of the latent behavioural classes’ recognition performed in RAZO_3 underwater, UW1 = Descending phase, UW2 = Searching/Catching phase, UW3 = Ascending phase.
