# Supplementary material for: The use of an unsupervised learning approach for characterizing latent behaviors in accelerometer data
Source: Ecol Evol. 2016 Jan 11;6(3):727–41. doi: 10.1002/ece3.1914 (PMC4739568; doi:10.1002/ece3.1914)
Supplement: Supplementary file 4 — Table S1. Transition probability matrix of the behavioural states classified in the 5 razorbills. Table S2. Transition probability matrix of the behavioural states classified in the 2 guillemots. [file ECE3-6-727-s003.doc]

Table 1: Transition probability matrix of the behavioural states classified in the 5 razorbills. UW1= Descending, UW2 = Searching/Catching, UW3= Ascending, All AW = all above water activities (high, medium and low activity, i.e. flying/flapping, floating, still at the colony).

| **RAZO** | **UW1** | **UW2** | **UW3** | **All AW** |
| --- | --- | --- | --- | --- |
| **UW1** | 0.98949 | 0.00445 | 0.00568 | 0.00039 |
| **UW2** | 0.00246 | 0.98703 | 0.00628 | 0.00423 |
| **UW3** | 0.00510 | 0.01012 | 0.97973 | 0.00103 |
| **All AW** | 0.00012 | 0.00013 | 0 | 0.99973 |

Table 2: Transition probability matrix of the behavioural states classified in the 2 guillemots. UW1= Descending, UW2 = Searching in the water column, COGU1 UW3= shallow activity, COGU2 UW3 = Deep Searching, UW4 = Chasing/Catching, UW5 = Ascending, All AW = all above water activities (high, medium and low activity, i.e. flying/flapping, floating, still at the colony).

| **COGU** | **UW1** | **UW2** | **COGU1**  **UW3** | **COGU2**  **UW3** | **UW4** | **UW5** | **All AW** |
| --- | --- | --- | --- | --- | --- | --- | --- |
| **UW1** | 0.99644 | 0.00153 | 0 | 0.00111 | 0.00092 | 0 | 0 |
| **UW2** | 0.00078 | 0.99194 | 0 | 0 | 0.00256 | 0.00218 | 0.00254 |
| **COGU1**  **UW3** | 0 | 0.00204 | 0.99488 | 0 | 0.00016 | 0.00141 | 0.00152 |
| **COGU2**  **UW3** | 0.00024 | 0 | 0 | 0.99754 | 0.00163 | 0.00058 | 0 |
| **UW4** | 0.00149 | 0.00392 | 0.00007 | 0.00105 | 0.99069 | 0.00239 | 0.00039 |
| **UW5** | 0 | 0.00391 | 0.00012 | 0.00012 | 0.00147 | 0.99386 | 0.00053 |
| **All AW** | 0.00002 | 0.00001 | 0.00001 | 0 | 0.00002 | 0 | 0.99994 |
